# Peer mentorship to promote effective pain management in adolescents: study protocol for a randomised controlled trial

**DOI:** 10.1186/1745-6215-12-132

**Published:** 2011-05-22

**Authors:** Laura B Allen, Jennie CI Tsao, Loran P Hayes, Lonnie K Zeltzer

**Affiliations:** 1 Pediatric Pain Program, David Geffen School of Medicine at UCLA, Los Angeles, CA, USA

## Abstract

**Background:**

This protocol is for a study of a new program to improve outcomes in children suffering from chronic pain disorders, such as fibromyalgia, recurrent headache, or recurrent abdominal pain. Although teaching active pain self-management skills through cognitive-behavioral therapy (CBT) or a complementary program such as hypnotherapy or yoga has been shown to improve pain and functioning, children with low expectations of skill-building programs may lack motivation to comply with therapists' recommendations. This study will develop and test a new manualized peer-mentorship program which will provide modeling and reinforcement by peers to other adolescents with chronic pain (the mentored participants). The mentorship program will encourage mentored participants to engage in therapies that promote the learning of pain self-management skills and to support the mentored participants' practice of these skills. The study will examine the feasibility of this intervention for both mentors and mentored participants, and will assess the preliminary effectiveness of this program on mentored participants' pain and functional disability.

**Methods:**

This protocol will recruit adolescents ages 12-17 with chronic pain and randomly assign them to either peer mentorship or a treatment-as-usual control group. Mentored participants will be matched with peer mentors of similar age (ages 14-18) who have actively participated in various treatment modalities through the UCLA Pediatric Pain Program and have learned to function successfully with a chronic pain disorder. The mentors will present information to mentored participants in a supervised and monitored telephone interaction for 2 months to encourage participation in skill-building programs. The control group will receive usual care but without the mentorship intervention. Mentored and control subjects' pain and functioning will be assessed at 2 months (end of intervention for mentored participants) and at 4 month follow-up to see if improvements persist. Measures of treatment adherence, pain, disability, and anxiety and depression will be assessed throughout study participation. Qualitative interviews for mentors, mentored participants, and control subjects will also be administered.

**Trial registration:**

ClinicalTrials.gov NCT01118988.

## Background

Chronic intractable non-malignant pain, including such functional disorders as recurrent abdominal pain (RAP) and chronic daily headache, is now recognized as a significant problem in children and adolescents, with potential long-term impact on the child's physical, social, and academic functioning, as well as on the family as a whole. Estimates of children experiencing recurrent or continuous pain range from 25% - 45.5%, with the most common types of pain reported as headaches, abdominal pain, limb pain, and back pain [1,2]. Many such children apparently continue to function effectively, attending school and continuing normal activities, with medical intervention only for acute episodes. A smaller, but significant, number, however, find themselves unable to self-manage their pain. They become patients with chronic pain and disability, falling into a cyclical pattern of pain, impaired functioning in physical, school, social, and even family and self-care domains, "doctor-seeking" and over-utilization of medications, and psychosocial distress, including anxiety and depression [3].

Functional impairment, particularly in academic work and social participation, is likely to have long-term effects on the individual's quality of life, even aside from the possibility that pain and physical limitations may persist into adulthood. Several well-designed studies using quantitative measures have provided evidence that impaired functioning in children with chronic pain is strongly associated with psychosocial distress [4-6] and with lower quality of life [4,7-10]. In particular, children with *unexplained *chronic pain - pain not associated with an identified organic diagnosis - often report significant dysfunction in normal activities, such as schoolwork, sleep, family activities, and athletic activities [10]. Although impaired functioning is a major factor in lower quality of life for children with chronic pain, we still know relatively little about the prevalence and severity of functional impairment, why some children experience more limitations than others, and which treatment interventions are the most effective in improving function [11].

### Current Therapeutic Interventions

When chronic pain cannot be fully alleviated, the optimal goal is for the adolescent is to learn effective ways to continue functioning and to self-manage pain. Several therapeutic programs have been developed, based on theories of health behavior change, to assist the patient in this process [12]. The newer therapeutic programs, the most well-known and widely practiced of which is cognitive-behavioral therapy (CBT), seek to mediate behavioral change through cognitive relearning. CBT employs a number of methods - including psychoeducation about the nature of pain responses, identification and modification of maladaptive cognitions, and encouraging behavioral changes to reduce or eliminate avoidance of activities [13,14]. Numerous controlled trials have shown CBT to be effective for a variety of types of pain, including in the child and adolescent populations [14-19].

In addition to psychotherapy, other types of complementary and alternative medicine (CAM) practices have shown varying levels of efficacy for chronic pain. These include interventions such as Iyengar yoga for rheumatoid arthritis [20], and acupuncture for low back pain [21]. In a 2005 review of studies examining CAM treatments for chronic pain conditions in children, Tsao and Zeltzer [22] found hypnosis and guided imagery to be "efficacious" for recurrent pediatric headaches, and found acupuncture, biofeedback, creative arts, herbal therapy, homeopathy, and massage therapy to be either "promising" or "possibly efficacious" for a wide variety of pediatric chronic pain conditions. Furthermore, the authors found multiple modalities packaged together to be more efficacious.

A well-designed intervention, however, is not enough. Patient *motivation *is at least one of the critical factors in successful outcomes of this therapeutic model [14]. Jensen and colleagues have recently proposed a cogent general model that integrates the varied theoretical approaches to describe a dynamic process that pivots on this concept of motivation, or readiness to change. An individual's readiness to change, they argue, is essential to his/her ability to learn successful pain self-management through new behaviors. They suggest some clinical approaches for enhancing readiness and promoting change, including encouragement to practice self-management; allowing the patient to observe other pain patients practice self-management; support of positive beliefs and non-judgmental non-support of negative beliefs; and development of a plan to address real or perceived barriers; and they call for research into interventions along these lines to enhance motivation [12].

Another formulation recently proposed by Sharp stresses the patient's cognitive activity in appraising and evaluating his or her pain, and its ongoing and interactive effects on mood, behavior, and somatic focus [23]. The patient's initial response to the pain is a function of cultural beliefs, learning history, and current contingencies, he argues, but then is continually reinterpreted with ongoing events. In particular, anxiety about recurrent pain and avoidance of activity that might cause pain will help to perpetuate the patient's hypervigilance for signs of recurring pain (as described by Eccleston and Crombez [24]) and his/her perceived inability to manage the pain. Moreover, Sharp contends that this attitude of "learned helplessness" may be perpetuated by physicians who have failed to offer helpful treatment or even to confirm the physical reality of the patient's suffering. "That is, patients could start to believe that 'nothing has worked so far so why would any future treatment help?" [23] A patient who has reached this point is likely to have a negative assessment both of the benefits of pain self-management and of his/her own ability or self-efficacy to learn these skills, and will therefore show a lack of readiness to change.

While current interventions such as those mentioned above are known to be effective, adherence to recommended treatments in children with chronic health conditions is typically low [25]. Simons et al. [19] report only a 46.7% rate of "full adherence" (defined as completing the full recommended course of treatment or currently receiving the treatment) to a referral of cognitive-behavioral therapy for children with chronic pain, lower than adherence rates for other treatment modalities (i.e., medical and physical therapy referrals). The authors also assessed barriers to treatment among non-adherents and found that negative attitudes and beliefs regarding the recommended intervention to be the most frequently cited reason for not completing the recommended treatment. This finding suggests that determining methods to improve adherence to recommended treatments is a necessary step to improving pain and functioning in this population.

### Strategies for Adolescents: Peer Mentoring

Innovative strategies to promote pain self-management in *adolescents *with chronic pain may be especially needed, as this age group has shown great variance in motivation and adherence in studies of CBT-based therapies for other chronic health conditions. In fact, children of all ages demonstrate low rates of adherence to recommended treatments for chronic health conditions, ranging from 11% to 50% [25]. However, some researchers have reported that motivation and adherence can be enhanced if young people have the opportunity to interact with peers who model and reinforce adherent behaviors, and Varni et al. have argued convincingly for "the power of peer relationships and social supports in mediating adjustment to chronic malfunctions" [25,26].

Peer mentors most often work in programs designed to encourage desired social and preventive health behaviors, such as nutrition and tobacco abstinence [27], non-violence [28], sexual abstinence [29], or HIV prevention [30] in physically healthy children. In a low-income area of Chicago, for example, 19 self-selected adolescents aged 14-21 designed and presented violence-prevention lessons to 50 younger children (7-13) over an 18-month period. Post-intervention, the mentored children showed lower acceptance of violence after the intervention than a control group of 75 children [28]; 11 of the adolescents continued with the program and 3 entered college or employment. Peer mentorship has only been explored in a few instances within medical treatment programs. In a youth-run program in San Francisco, peer-mentor-advocates have effectively empowered young people infected with HIV (ages 26 and younger) to access needed services and to learn coping skills for living with the disease [31]. Peer support has also been the subject of several studies of adolescents with diabetes, where researchers have found that the support of friend-peers is a critical resource for diabetic teens, providing important social and emotional support, and that structured peer-group interactions *may *improve adherence to glucose self-monitoring and control [32-34]. Greco and colleagues reported a successful education and support group intervention with 21 diabetic adolescents (ages 10-18) and their best friends (4 sessions); both the patients and their friends demonstrated higher levels of knowledge about the disease and its care, and the patients reported a higher ratio of peer-to-family support [33]. Clemente has used ethnographic methods to document the informal peer-mentoring provided to children newly admitted to cancer treatment wards by peers who had been through several treatment cycles [35]. We contend that trained peer mentors, who have successfully learned pain self-management skills, are an effective and feasible choice to promote pain self-management in the adolescent chronic pain population because this intervention has the potential to relieve the sense of isolation, difference, and helplessness reported by the adolescents.

In this exploratory pilot study, we will finalize development of an innovative, manualized peer-mentorship program designed to provide modeling and reinforcement by peers to other adolescents with chronic pain (the "mentored participants"). The goal of the mentorship will be to encourage the mentored participants to engage in therapies that promote the learning of pain self-management skills and to support the mentored participants' practice of these learned skills during a two-month intervention period. The study will examine the feasibility of this intervention for both mentors and mentored participants, and, through a randomized, controlled design, will assess the preliminary effectiveness of this program for both short and longer term effects on mentored participants' pain and functional disability.

### Aims

1. To test the feasibility and acceptability of a peer-mentorship intervention in supporting and encouraging adolescents with chronic pain and pain-related functional disability (the "mentored participants") to participate and persist in an active skills-building therapy that teaches pain self-management skills.

2. To test the feasibility of training adolescents who have coped successfully with chronic pain (the "mentors") to offer support and reinforcement to their peers in a structured peer-mentorship intervention, based on a manualized protocol developed within a social learning model grounded in CBT principles.

3. To determine if the mentored participants with chronic pain show improved adherence to recommended skills-building therapies, compared to a usual care control group of adolescents with chronic pain immediately after the two-month peer-mentorship intervention and at two-month follow-up (four months from baseline).

4. To determine if the mentored participants with chronic pain show improvements in pain levels and pain-related disability, compared to a usual care control group of adolescents with chronic pain immediately after the two-month peer-mentorship intervention and at two-month follow-up.

### Hypotheses

We propose to test our hypotheses through a trial of a peer mentorship intervention, using trained adolescents who have successfully learned pain management skills as mentors. The mentors will help to relieve the adolescents' sense of isolation and difference by relating their similar experiences, provide models of successful skill learning and reinforce the mentored participants' participation in skill learning activities. We propose the following hypotheses:

1. Adolescents receiving the peer mentorship intervention will report better adherence to recommended therapies, as compared to adolescents who do not receive the intervention.

2. Adolescents with better adherence to recommended therapies will show more improvement in pain and pain-related disability 2 and 4 months after baseline than those with poor adherence.

3. Adolescents receiving the peer mentorship intervention will report decreased symptoms of anxiety and depression and improved pain coping skills, as compared to adolescents who do not receive the intervention.

## Methods/Design

### Sample

#### Mentored and Control Subjects

All potential participants must be patients of the UCLA Pediatric Pain Program. During the first visit (prior to being offered study participation), all clinic patients are given a thorough medical evaluation and are recommended specific therapies to help with various skills to manage their pain. Any English-speaking patient between the ages of 12 and 17 diagnosed with a chronic pain disorder at the UCLA Pediatric Pain Program, or referred with this diagnosis, is eligible to be a subject for the study. Participants must have access to a computer connected to the internet, as well as a telephone in order to participate in the mentoring and complete measures administered online. Other than age, the only exclusions are out-of-area patients seen for consultation only before referral back to local providers or patients with another physical or psychological condition that, in the judgment of the principal investigator (PI), would make it difficult to participate.

#### Mentors

Any English-speaking patient between the ages of 14 and 18 years who has been treated for a chronic pain disorder at the UCLA Pediatric Pain Clinic and has been actively involved in recommended therapies will be eligible to serve as a mentor. Participants must have access to a computer connected to the internet, as well as a telephone. The PI (LKZ) will identify suitable mentors based on her assessment of the adolescent's maturity, emotional stability and verbal communication skills. Mentors will be compensated $12/session.

### Randomization

Participants recruited for the mentored or control portion of the study will be randomized using a block randomization procedure to assure equal sample sizes in the mentoring and control groups. Using this method allows the groups to have similar numbers of participants in each group at all times. Using a block size of 4, participants are assigned to the appropriate treatment condition as they enroll in the study until the block is completed. Then the following 4 participants are assigned based on the next block [36].

### Procedures

#### Mentor Training

For overall schematic of the procedures for mentor training and administration of the intervention see Figure 2. Mentors will receive six hours of training with trained doctoral-level research study personnel in the UCLA Pediatric Pain Program prior to working with mentored participants.

##### Training manual

The training material will include background on chronic pain disorders and rationales for pain self-management; specific goals of the program and the mentor's role; how to present information about pain and pain-related behaviors; strategies drawn from CBT to reinforce positive thinking and to challenge negative statements; guidelines for building the mentoring relationship, dealing with difficult issues (e.g., depression, suicidality, or other potential emergencies such as disclosure of abuse), and ending the interactions. Mentors will be given a written manual presenting all the material in detail for their ongoing reference. The mentors' specific objectives will be to provide information about pain, pain-related behaviors, thoughts, and feelings, and the nature of recommended treatments, as well as to alleviate the subject's sense of isolation by giving him or her the opportunity to discuss his or her pain condition with someone who has shared the experience; to enhance and reinforce the subject's sense of self-efficacy to manage the pain condition; and to encourage the mentored participant to participate actively in the recommended pain self-management skills building therapy. Mentors will be trained in conversational strategies to help them meet the objectives without being overly directive (see details below).

##### Peer Mentor Training

A postdoctoral psychologist (LBA) together with other research staff trainers will begin conducting training when at least six (6) peer mentors have been identified and recruited. Training will consist of one six-hour session including all mentors, which will include the following: 1) Review of slide presentation and rationale for learning pain self-management, as well as the contribution of biological, psychological, and social factors to the pain experience; 2) demonstration and practice of some basic skills to use in working with the mentored participants; 3) review of the manual; outline of the format and required elements for each call; 4) participation in role-playing exercises in pairs to learn ways of presenting didactic information, supporting positive statements, and responding to questions posed by the mentored participant; 5) participation in question development and topic-starter exercises; 6) participation in troubleshooting exercises to suggest ways of addressing particular problems; 7) review of call formats, reporting requirements and detailed discussion of emergency situation procedures.

Prior to working with actual study patients, the best surrogates for the mentors to practice their new skills with are their fellow mentors, who will all be adolescents with resolved or ongoing recurrent pain problems, whose recent experiences have been similar to those of the mentored participants. Each mentor will participate in several different role-playing exercises during training, in which the mentors will break into pairs and improvise conversations that will occur between mentor and mentored participant. For example, they might be told: "One of you will be the mentored participant who has been to a couple of therapy sessions, but is feeling discouraged, like therapy isn't working and probably nothing is going to help. Talk to your partner and choose a specific therapy. Make up a conversation between the two of you, in which the mentored participant wants to give up on therapy and the mentor responds to those feelings. Remember what you have learned about cognitive-behavioral principles in thinking about what the mentor should say." These mock conversations will be observed and critiqued by trainers. Each mentor will be assigned to no more than three mentored participants at any one time; some mentors will only work with one mentored participant during some cycles, to ensure they can take on a mentored participant if one of the group becomes ill or has another emergency. (Additional mentors may be trained during the intervention period if needed due to attrition or higher than anticipated subject enrollment.) During the period of active mentorships (about 18 months), mentors will meet monthly with the research team to review guidelines, provide feedback, and discuss problems that may arise. Mentors will participate in the ongoing iterative process of reviewing and refining the protocol after each cycle.]

##### Emergency Support

Particular attention will be paid to the rare emergency situations that may arise in the course of mentor-mentored participant conversations, such as disclosure of domestic abuse, suicidal or self-harm intent. Procedures will be discussed at length in the training session. The mentor will be given detailed instructions about how to behave in these specific situations. In addition, parents of peer mentors will agree to be reachable at all times either in person or by phone in case of emergency. Following any such occurrence, the mentor will be given immediate counseling by the PI or a monitoring research team member, and will be referred for extended counseling if s/he suffers resulting distress. In addition, all calls will be monitored directly by a member of the research team trained in emergency procedures (i.e. for abuse, suicidality disclosures, etc.) and recorded using digital audio recorders.

### Mentored/Control Subject Assessment

**Figure 1:**
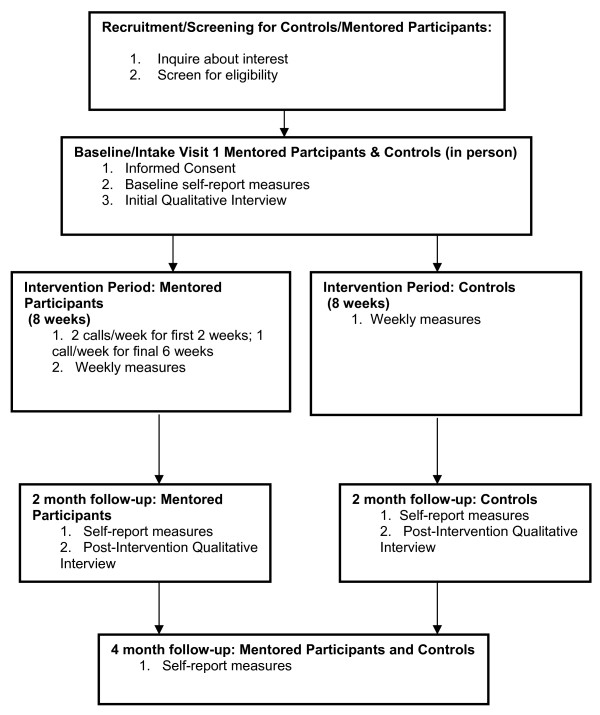
**Procedural flow chart for mentored participants and controls**.

For an overall schematic of the assessment schedule for mentored participants and controls, please see Figure 1. The baseline and 2-month follow up assessments will consist of a semi-structured interview and self-report questionnaires. The final assessment at four months from baseline consists only of a self-report questionnaire packet. For the mentored participants, the study will consist of baseline assessment, two months of active intervention, immediate follow-up assessment at the end of intervention, and then a final assessment at four months from baseline. For the controls, the study will consist of the baseline assessment, two months of usual care, immediate assessment, and then follow-up assessment at four months.

**Figure 2:**
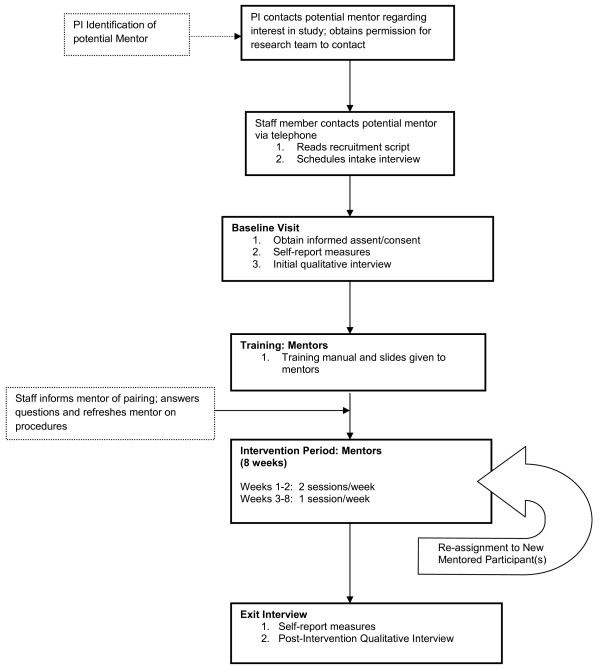
**Procedural flow chart for mentors**.

Mentored participants and control subjects will be called once per week during the intervention/usual period (first 8 weeks of the study) by trained research study personnel to assess use of recommended therapies, medications, and additional non-physician recommended therapies. During these weekly assessments, research staff will also assess for the presence of any suicidal ideation/intent. In addition to monitoring suicidality, several other assessment measures focused on pain, disability, and negative affect will be completed weekly by mentored participant and control subjects online using a web-based program from a computer in their homes.

### Intervention

Each mentored participant will be assigned to a peer mentor. Each mentor will work with no more than three subjects at one time; once all mentors have been assigned one to three subjects, recruitment will stop until the intervention cycle concludes. Cycles may be staggered depending on recruitment and mentor availability.

Each cycle will consist of 10 sessions of 30-60 minute phone calls conducted over a period of eight weeks. The mentor and mentored participant(s) will have two sessions per week for the first two weeks, and one session per week for the remaining six weeks. Current literature supports the use of an expanding-interval schedule of interventions (compared to a massed (single session) or uniform-interval schedule) with evidence that the expanding schedule may help to attenuate long-term recurrence of negative emotions [37]. Phone calls will take place on a secured, monitored conference call line so that no participants will exchange personal contact information. The time of all phone meetings will be scheduled by the research staff, based on availability of mentor and mentored participant(s) and will remain at a fixed time each week. Mentoring sessions will occur only during the scheduled times and mentored participant-mentor contact outside of the mentoring sessions will be specifically prohibited. During each session, participants will also be viewing a slide presentation at the time of the conference call, so that didactic information can be presented. This presentation has been developed by doctoral-level research staff. As previously mentioned, to ensure safety of all participants, a member of the research team will be monitoring all telephone calls. This individual will facilitate the discussion, only if necessary, as well as intervene in any potential safety concerns (e.g., discussion of suicidal ideation) or inappropriate behavior (e.g., bullying).

In an effort to increase adherence, mentored participants will be informed that if they miss two consecutive mentoring sessions, or miss three sessions total over the course of the intervention for non-emergency reasons, they will be removed from the study. Additionally, peer mentors who miss two mentoring sessions (for non-emergency reasons) will be removed from further participation as a mentor. Mentored participants will then be reassigned to a new mentor for the remaining sessions.

### Ethics

All participants (mentors, mentored participants and control subjects) provide voluntary written informed assent or consent with full knowledge of study participation requirements and potential risks. When required for participants who are legal minors, voluntary written informed parental consent is also requested. All participants are informed that any decision about participation will have no effect on their relationship with UCLA or their medical care at UCLA. This study was approved by the UCLA Institutional Review Board.

### Measures

#### Primary Outcomes

1. ***Adherence ***to recommended therapies, use of additional therapies, and medication use will be evaluated using the Treatment Services Tracking Form - a measure designed specifically for use in this study to assess number of visits and missed appointments with team clinicians each week. This form is administered verbally by research staff. Treatment adherence reported by participants is then corroborated with reports from program clinicians.

2. ***Pain: ***Demographics questionnaire and pain level as indicated on a body diagram and several Numerical Rating Scales (NRS; 0-10 scale) [38] to represent a continuum from no pain at all to the worst pain possible. The NRS has been established and widely used as a valid and reliable measure of pain intensity.

3. ***Functioning:***

a. Self-perceptions of difficulty in physical and psychosocial functioning due to physical health as measured on the Functional Disability Inventory (FDI) [39], a well-validated 14-item self-report questionnaire with a five point scale that asks children the level of difficulty they have with typical daily activities.

b. Self-perceptions of health and functioning as measured on the Children's Health Questionnaire (CHQ), a quality of life instrument designed for use with children age 5 and above. The CHQ measures 14 unique physical and psychosocial health concepts (e.g., physical functioning, limitations in school, self-esteem, mental health, family functioning) that can be analyzed and reported separately or combined to derive an overall physical and overall psychosocial score. There are 87 items [40].

#### Secondary Outcomes

4. ***Anxiety and Depressive Symptoms:***

a. The Child Anxiety Sensitivity Index (CASI), an 18-item scale for measuring anxiety sensitivity. Children rate each item (e.g., It scares me when I feel like I am going to throw up) on a scale ranging from *none *to *a lot *[41].

b. Symptoms corresponding to selected *DSM-IV *anxiety disorders and depression, as assessed by the 47-item, 4-point, Revised Child Anxiety and Depression Scale (RCADS) [42,43].

c. Suicidal ideation will be assessed using a question from the Beck Depression Inventory - 2^nd^ edition (BDI-II) to assess suicidality [44].

For a complete list of measures and time points at which they are administered for each subject group, please see Tables 1 and 2.

**Table 1:** Timetable for study participation for mentored and control subjects

	TIME PERIOD					
	
STUDY PARTICIPATION FOR ADOLESCENT MENTORED AND CONTROL SUBJECTS	Baseline	Week 1-2	Week 3-8	2-month follow-up	Weeks 9-16	4-month follow-up
Measures						

Demographics, NRS & body diagram	X	X	X	X		X

FDI	X	X	X	X		X

CASI	X			X		X

RCADS	X			X		X

BDI-II suicidality	X	X	X	X		X

Treatment tracking	X	X	X	X		X

Interview	X			X		

Estimated Time Required for Study Participation	76 MINS	140-260 MINS	240-420 MINS	86 MINS		56 MINS

**Table 2:** Timetable for study participation for peer mentors

	TIME PERIOD				
**STUDY PARTICIPATION FOR ADOLESCENT PEER MENTORS**	**Months 1-2**	**Months 3-4 (first cycle)**	**Month 5 (break)**	**Months 6-18 (cycles 2-5)**	**Month 19**

Training Sessions	X				

Mentorship Phone Talks and Debriefings		X		X	

Questionnaire Packet	X				X

Assessment and Review Meetings		X		X	

Interview	X				X

Estimated Time Required for Study Participation	7.2-7.8 HOURS	9 HOURS		36 HOURS	70-100 MINS

### Statistical analysis

#### Sample size

##### Sample Size Determination and Power Analysis

The proposed sample size was calculated with two objectives in mind: 1) to remain realistic in terms of recruitment based on our anticipated patient flow and the proposed time frame; and 2) to ensure the power to detect significant group differences in the primary outcome measures. Based on our average patient intake of 8-12 new patients a month and the parameters of our existing population, we estimate that we will be able to recruit 3-4 per month in the target age group (12-17) to participate in this pilot study, or a minimum *N *of 50. About 70% will be girls, 30% boys. The majority will be non-Hispanic white, but about 33% will be from other ethnic groups or of mixed-race. The second objective in calculating the proposed sample size is to ensure adequate power (.80) to detect variable relationships (two-tailed, *p *< .05) when conducting the primary analyses. Since there have been no comparable studies of peer mentorship interventions and there are no data available to calculate effect sizes for adherence to skill-based therapies in a pediatric chronic pain population, we have chosen the conservative strategy of extrapolating from other studies of pediatric pain that have used the Functional Disability Inventory (FDI) [39] as an outcome measure in evaluating a CBT-based intervention, in making our power calculations. One study [45] included 30 adolescents with fibromyalgia randomized to either 8 weeks of coping skills training or control (self-monitoring) and then crossed-over to the alternate condition. For the FDI, the authors reported overall effect sizes of 1.29 for patients (n = 14) who received the self-monitoring first and then the coping skills training and 0.52 for patients (n = 13) who received the coping skills training followed by self-monitoring. A second study examined an 8 week CBT trial in 44 adolescents with fibromyalgia [46]. These authors found a significant reduction in the FDI following treatment compared to waitlist control, with an effect size of 0.75. Using the smallest effect size reported in these two studies (i.e., .52), which corresponds to a medium sized effect according to the conventions proposed by Cohen [47], with power of .80 to detect a significant difference in FDI scores using two-tailed tests and with alpha set at .05, it is estimated that we will be able to detect this effect size with 29 subjects per group.

We expect to recruit 4 - 6 mentored participants for each two-month cycle during the active intervention period of 16 months, yielding approximately 32 - 48 mentored participants, with a corresponding number of controls, and seven mentors, giving a total study enrollment number of 70 - 100.

##### Preliminary Analyses

Once the data set has been cleaned and examined for skewness and missing data, exploratory data analyses will be conducted using univariate methods to describe the variables of interest and bivariate techniques to characterize their interrelationships within our sample. Continuous variables will be summarized over time in each group using means and medians and we will also report their range, interquartile range and standard deviations. Cross tabulated frequencies will be given for all discrete variables by group and time. Both parametric and non parametric correlations will be reported. Results from these descriptive analyses will be used to help guide the primary analyses by providing information about potential problem measures (e.g., heavily skewed distributions) or problematic variable relationships (e.g., highly correlated predictors) and will help determine if transformations of some outcomes (such as percent adherence) are possible in order to use parametric methods. Only after a full descriptive investigation of the dataset has been achieved and potential problem variables identified will primary analyses commence. Potential covariates will be examined prior to final statistical modeling to determine whether there are pre-existing differences between the groups. These exploratory analyses will test for differences in demographic and clinical parameters as well as psychological aspects that have been found to correlate with functioning in our prior work. We will also look at mentored participant outcomes within and across mentors to attempt to determine if there are any discernable patterns of improvement or decline in functioning as a function of clustering of outcomes within mentors or outcomes as a function of each mentor's experience with the protocol. This will help account for therapist effects as well as potential learning and/or fatigue effects. Any variables found to vary significantly by group will be included as covariates in primary and secondary analyses. This will also serve to verify that the randomization was successful.

##### Primary Analyses

The primary outcomes are adherence (percent of sessions attended, percent of home exercises completed). The primary analysis method is repeated measure analysis of variance (ANOVA) between the two groups or the non parametric equivalent (Friedman procedure). We will subtract baseline value from the 2- and 4-month assessment values and use residualized change scores from baseline as the outcome. We will use the Tukey-Fisher criteria for post hoc mean/median comparisons under the ANOVA or Friedman model. The same methods will be used for the analyses on NRS and FDI scores.

##### Secondary Analyses

Repeated measure analysis of variance (ANOVA) between the two groups or the non parametric equivalent (Friedman procedure) will also be used to evaluate differences in anxiety and depressive symptoms (RCADS, CASI) and additional measures. We will also look at the relation between session attendance (percent sessions attended) and home exercise completion (percent exercises completed) versus FDI change from baseline. The relation will be quantified by the Pearson and Spearman correlations. If appropriate, we will also use regression methods and report intercepts and slopes.

##### Attrition

We will compare attrition rates between the two treatment groups and compare demographics and baseline values of FDI and other measures of dropouts to non-dropouts. If dropouts are random, then the results are unbiased. If dropouts are not at random, we will comment on the direction and magnitude of potential biases.

##### Interview Analysis

The major goal of the interview analysis is to elicit the mentor's and mentored participant's perceptions of the intervention and specific ways in which they found it helpful (or not helpful). Approximately 60 minutes will be recorded with each mentor and selected subject, for a total of 18 interview hours. The interviewer will use a semi-structured script suggests topic areas and questions, but allows adolescents to develop each topic in their own words. All interviews will be transcribed and coded using N*6 software and applying the principles of grounded theory [47,48].

The coders will code each interview for the number and strength of the each of the following types of statements: 1) statements of functioning limitations *or *of functioning abilities; 2) statements of self-efficacy to achieve goals *or *of lack of self-efficacy; 3) statements that mentorship was helpful *or *that it gave little help; 4) statements that learning a pain self-management skill was helpful *or *of little help. The *strength *of a statement will be indicated by the presence of modifiers and will be valued using a table of standard statements. Examples of statements in order of increasing strength: "Mom says I should be able to walk a mile now," "I think I can walk a mile now." "I know I could walk a mile now," "I walked a mile yesterday," "I often walk a mile now." Values of statements regarding functioning and self-efficacy will be compared with changes on quantitative measures for each child to see if the different types of data corroborate each other; and also compared with the values of the child's statements regarding helpfulness of the interventions. Statements regarding the helpfulness of the peer mentorship intervention and skills building therapies will be evaluated for strength and used in the evaluation of the program.

Additional context-based codes may be assigned, using the constant comparison method [49], to identify statements regarding treatment expectations and beliefs, pain expectations and beliefs, fears and anxieties. Coders will carry out this second-level coding to identify specific factors promoting changes in the child's functionality from intake through the mentorship intervention. All statements then will be analyzed to determine if the children's accounts support the Jensen model [12] of behavior change described above.

## Discussion

This is an exploratory pilot study in which good evidence of outcome intervention will depend on the success of a mentor training program which is still in development. We have tried to plan all aspects of recruitment and training carefully, but mentor retention and mentor adherence to a structured intervention protocol may vary with factors we have not considered. We will be evaluating carefully the scheduling, format, and content of communications, as well as the attrition rate for the control group at both assessment timepoints. We considered using electronic communication because of its greater flexibility, but phone communication was selected because it will promote social interaction for those patients who may have stopped attending school and other activities outside the home. Peer group therapy is another alternative to introduce peer modeling and reinforcement into a treatment program to improve functioning; but our experience suggests that group meetings often prove impractical for adolescents and families with busy daily schedules. Sample size has been estimated based on other studies of pediatric pain that have evaluated CBT-based interventions and reported a large effect size. We may not have the same effect size and may have underpowered this pilot study. Finally, some patients' participation in recommended therapies may be limited by factors, such as insurance authorizations, over which we have no control.

This exploratory pilot study will enable us to refine the features of the peer-mentorship model - mentor training, structure and content of the manual and protocol, length and format of interactions - and to test our hypotheses that mentors and mentored participants will find this intervention acceptable and that mentored participants will demonstrate improved functioning in comparison to a usual care control group by follow-up. These findings will be disseminated in papers submitted to peer-reviewed journals. We then plan to test the refined model in a controlled R01 trial with a larger sample and longer-term subject follow-up, with a mediator/moderator model.

## List of Abbreviations Used

ANOVA: Analysis of variance; BDI-II: Beck Depression Inventory: 2nd edition; CASI: Child Anxiety Sensitivity Index; CBT: Cognitive-behavioral therapy; CAM: Complementary and alternative medicine; FDI: Functional Disability Inventory; NRS: Numerical Rating Scales; PI: Principal investigator; RAP: Recurrent abdominal pain; RCADS: Revised Child Anxiety and Depression Scale.

## Competing interests

The authors declare that they have no competing interests.

## Authors' contributions

JCIT and LKZ participated in the conception of the trial and in plans for the data analysis, with additional input from LBA. LBA, JCIT, LPH and LKZ drafted the manuscript. All authors read and approved the final manuscript.
